# A Surgeon-Centered Neuromuscular Block Protocol Improving Intraoperative Neuromonitoring Outcome of Thyroid Surgery

**DOI:** 10.3389/fendo.2022.817476

**Published:** 2022-02-10

**Authors:** I-Cheng Lu, Chiung-Dan Hsu, Pi-Ying Chang, Sheng-Hua Wu, Tzu-Yen Huang, Yi-Chu Lin, How-Yun Ko, Gianlorenzo Dionigi, Young Jun Chai, Feng-Yu Chiang, Yi-Wei Kuo, Che-Wei Wu

**Affiliations:** ^1^Department of Anesthesiology, Kaohsiung Municipal Siaogang Hospital, Kaohsiung Medical University Hospital, Kaohsiung Medical University, Kaohsiung, Taiwan; ^2^Faculty of Medicine, College of Medicine, Kaohsiung Medical University, Kaohsiung, Taiwan; ^3^Department of Anesthesiology, Kaohsiung Municipal Ta-Tung Hospital, Kaohsiung Medical University Hospital, Kaohsiung Medical University, Kaohsiung, Taiwan; ^4^Department of Otorhinolaryngology, Kaohsiung Medical University Hospital, Kaohsiung Medical University, Kaohsiung, Taiwan; ^5^Division of General Surgery, Endocrine Surgery Section, Istituto Auxologico Italiano IRCCS (Istituto di ricovero e cura a carattere scientifico), Milan, Italy; ^6^Department of Pathophysiology and Transplantation, University of Milan, Milan, Italy; ^7^Department of Surgery, Seoul National University College of Medicine, Seoul Metropolitan Government—Seoul National University Boramae Medical Center, Transdisciplinary Department of Medicine & Advanced Technology, Seoul National University Hospital, Seoul, South Korea; ^8^Department of Otolaryngology, E-Da Hospital, I-Shou University, Kaohsiung, Taiwan; ^9^Department of Anesthesiology, Kaohsiung Medical University Hospital, Kaohsiung Medical University, Kaohsiung, Taiwan

**Keywords:** intraoperative neuromonitoring (IONM), thyroid surgery, recurrent laryngeal nerve (RLN), neuromuscular block degree, sugammadex

## Abstract

**Background:**

Neuromuscular blocking agents provide muscular relaxation for tracheal intubation and surgery. However, the degree of neuromuscular block may disturb neuromuscular transmission and lead to weak electromyography during intraoperative neuromonitoring. This study aimed to investigate a surgeon-friendly neuromuscular block degree titrated sugammadex protocol to maintain both intraoperative neuromonitoring quality and surgical relaxation during thyroid surgery.

**Methods:**

A total of 116 patients were enrolled into two groups and underwent elective thyroid surgery with intraoperative neuromonitoring. All patients followed a standardized intraoperative neuromonitoring protocol with continuous neuromuscular transmission monitoring and received 0.6 mg/kg rocuronium for tracheal intubation. Patients were allocated into two groups according to the degree of neuromuscular block when the anterior surface of the thyroid gland was exposed. The neuromuscular block degree was assessed by the train-of-four (TOF) count and ratio. Patients in group I received sugammadex 0.25 mg/kg for non-deep neuromuscular block degree (TOF count = 1~4). Patients in group II were administered sugammadex 0.5 mg/kg for deep neuromuscular block degree (TOF count = 0). The quality of the intraoperative neuromonitoring was measured using the V_1_ electromyography (EMG) amplitude. An amplitude less than 500 μV and greater than 500 μV was defined as weak and satisfactory, respectively.

**Results:**

The quality of the intraoperative neuromonitoring was not different between groups I and II (satisfactory/weak: 75/1 vs. 38/2, *P* = 0.14). The quality of surgical relaxation was acceptable after sugammadex injection and showed no difference between groups [55/76 (72.3%) in group I vs. 33/40 (82.5%) in group II, *P* = 0.23].

**Conclusions:**

This surgeon-centered sugammadex protocol guided by neuromuscular block degree (0.5 mg/kg for deep block and 0.25 mg/kg for others) showed comparably high intraoperative neuromonitoring quality and adequate surgical relaxation. The results expanded the practicality of sugammadex for precise neuromuscular block management during monitored thyroidectomy.

## Introduction

Intraoperative neuromonitoring (IONM) has gained increasing popularity during thyroid surgery in recent years. IONM is an adjunct tool for the identification and localization, detecting anatomical variations, differentiating mechanisms of injury, and predicting the postoperative function of the recurrent laryngeal nerve (RLN) (1–12). The goal of IONM is to diminish the occurrence of RLN injury and vocal cord paralysis associated with thyroid and parathyroid surgery.

Both good contact of surface electrodes (13–19) and adequate reversal of neuromuscular blockade (NMB) (20–27) are prerequisites for successful IONM of the RLN. For adequate relaxation to facilitate tracheal intubation and surgery, a profound or deep NMB is desirable. A proper reversal of the NMB is mandatory for functional IONM *via* evoked electromyogram (EMG) signals. Recently, sugammadex following rocuronium during anesthesia induction has been reported as an effective NMB regimen for successful IONM in animal and human clinical studies involving thyroid surgery (28–30).

Various sugammadex regimens have been reported. Low-dose sugammadex can enhance spontaneous neuromuscular functional recovery, while high-dose sugammadex can result in undesirable involuntary movement during surgery. In our previous report, sugammadex at a dose of 0.5 mg/kg induced NMB reversal from rocuronium 0.6 mg/kg and provided high-quality monitoring of the RLN for thyroid surgery (31). Chai et al. (32) demonstrated that sugammadex at either 1 or 2 mg/kg resulted in high-quality IONM. The bucking effect of sugammadex is dose-related, such that 2 mg/kg sugammadex was associated with up to 35% higher effects than 1 mg/kg sugammadex (32). However, the titration of sugammadex dosage according to the degree of NMB has not been investigated.

This study aimed to establish a surgeon-friendly protocol by titrating the sugammadex dose based on the degree of NMB before initial vagus nerve stimulation (V_1_) to maintain both surgical relaxation and IONM quality during thyroid surgery. We hypothesized that sugammadex titrated at a dose of 0.25 mg/kg is effective for the reversal of rocuronium-induced moderate NMB and can provide effective IONM with sufficient surgical relaxation. We compared this protocol with 0.5 mg/kg sugammadex for deep NMB, which was routinely administered at our institution.

## Methods

### Patient Data

This retrospective observational study was approved by the institutional review board of Kaohsiung Medical University Hospital [KMUHIRB-E(I)-20210070] and registered at ClinicalTrials.gov (NCT 04982185). Patients who underwent elective total thyroidectomy or total lobectomy with routine IONM were included between August 1, 2019, and July 31, 2020. Patients who met the following exclusion criteria were excluded from the study: age ≤20 years, American Society of Anesthesiologists (ASA) status of ≥4, vocal cord palsy, or previous thyroid surgery. All operations were performed by the same surgeon, and anesthesia was administered by two experienced anesthesiologists. The neuromonitoring setup, surgical procedures, and loss of signal algorithm followed the International Neural Monitoring Study Group Guidelines (1, 5, 11, 12).

### Anesthesia

Upon arrival at the operating room, each patient was placed under standard physiological monitoring (oximetry, electrocardiography, non-invasive blood pressure, and capnography). Before anesthesia induction, a donut pad beneath the neck was placed for thyroidectomy. An oral endotracheal tube with a 7.0- and 7.5-mm internal diameter was placed for female and male patients, respectively.

The NMB degree was continuously monitored by the train-of-four (TOF) count and ratio derived from the adductor pollicis muscle. Anesthetic depth was assessed using the response entropy (RE) or bispectral index (BIS). Anesthesia induction was initiated with fentanyl (1 μg/kg), lidocaine (1 mg/kg), and propofol (1.5–2 mg/kg). When loss of consciousness was identified, rocuronium (0.6 mg/kg) was administered to induce NMB in all patients. When the maximum NMB was achieved, the anesthesiologist performed tracheal intubation using a Trachway video intubating stylet (Biotronic Instrument Enterprise Ltd., Tai Chung, Taiwan). The position of the endotracheal tube was confirmed by end-tidal CO_2_ and auscultation.

Anesthesia was maintained with sevoflurane and propofol target-controlled infusion with an Orchestra™ infusion pump (Fresenius Vial, France). The effect-site concentration of propofol was maintained at 1–1.5 µg/ml. Two registered nurse anesthetists followed the standardized anesthesia regimen according to our institution’s protocol and adjusted the inhaled sevoflurane concentration to maintain an entropy or BIS value between 40 and 60. A bolus of fentanyl 0.5 µg/kg was administered before the skin incision.

### Study Flowchart

**Figure 1:**
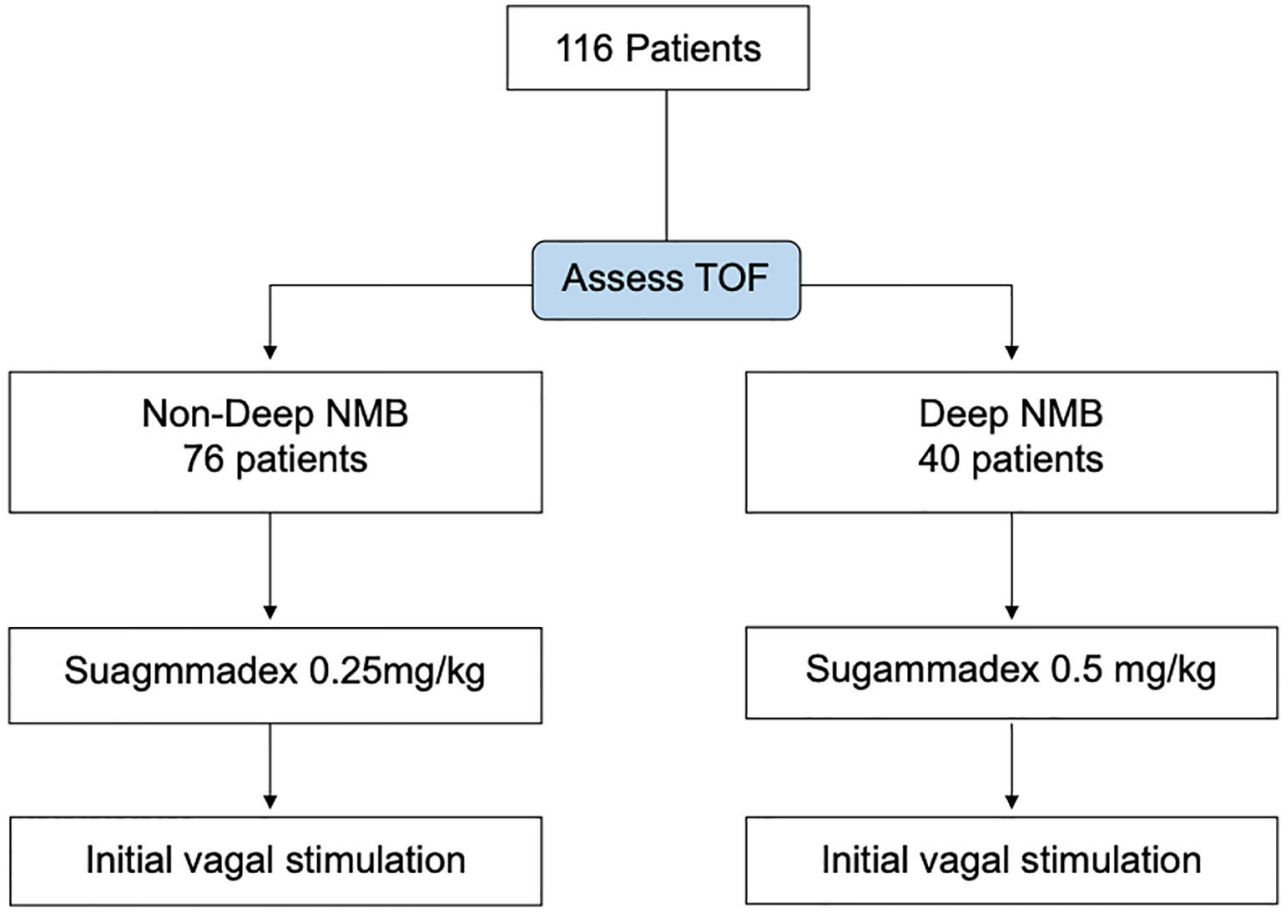
Study flowchart of the 116 consecutive patients, 40 with deep neuromuscular block (NMB) are reversed by sugammadex 0.5 mg/kg. Another 76 patients with moderate NMB or recovery are reversed by sugammadex 0.25 mg/kg. TOF, train of four.

The timing of sugammadex administration was decided by the surgeon after the initial surgical steps of subplatysmal flap creation and strap muscle separation to expose the anterior surface of the thyroid gland. Patients were allocated into two groups according to the degree of NMB before sugammadex administration. A TOF count of 0 was defined as deep NMB. A TOF count of 1–3 was defined as moderate neuromuscular block and 4 as recovery (33). Patients with non-deep NMB (group I) received sugammadex 0.25 mg/kg. Patients with deep NMB (group II) were administered sugammadex 0.5 mg/kg (**Figure 1**). All patients followed the standard anesthesia protocol for monitored thyroidectomy (**Table 1**).

**Table 1:** Departmental anesthesia protocol for intraoperative neuromonitoring during thyroid surgery.

Time courses	Remarks
**Preoperative evaluation**	ASA physical status and upper airway management assessment
**Monitoring setup**	Standard physical/neuromuscular/anesthesia depth monitors
**Anesthesia induction**	
Induction	Fentanyl 1 µg/kg, lidocaine 1 mg/kg, and propofol 1.5–2 mg/kg
NMBA	Rocuronium 0.6 mg/kg
**Anesthesia maintenance**	Avoid NMBA
Inhaled anesthetic	Sevoflurane 1%–4%
Intravenous anesthetic	Propofol TCI, effect-site concentration: 1~1.5 µg/kg
Anesthesia depth	Entropy or BIS between 40 and 60
Vasopressor	Ephedrine 8–10 mg if MAP reduction >20% mmHg
PONV prophylaxis	Dexamethasone 5 mg, avoid morphine
Inadequate relaxation	A bolus of fentanyl 0.5 µg/kg and propofol 20–30 mg
**Neural monitoring**	Low dose of sugammadex blockade
Placing TC electrodes	Sugammadex 0.5 mg/kg if TOF count = 0 (con)
	Sugammadex 0.25 mg/kg if TOF count = 1–4
V_1_ and V_2_ signal	EMG amplitude correlated with TOF ratio
**Anesthesia emergency**	
Extubation	Additional sugammadex up to 2.0 mg/kg
	Extubation when spontaneous breath with TOF ratio >0.95
Pain control	Parecoxib 40 mg or NSAID if not contraindicated
	Fentanyl 0.5 µg/kg
**Postoperative visit**	Anesthesia adverse events and satisfaction

ASA, American Society of Anesthesiologists; NMBA, neuromuscular blocking agent; TCI, target-controlled infusion; MAP, mean arterial pressure; BIS, bispectral index; PONV, postoperative nausea vomiting; TC, thyroid cartilage; V_1_ and V_2_, initial and final vagal stimulation; TOF, train-of-four mode of neuromuscular transmission monitoring; EMG, electromyography; NSAID, non-steroidal anti-inflammatory drug.

### Surgical Techniques and Intraoperative Neuromonitoring Setup

A lower neck skin incision was made along the skin crease. Subcutaneous fat and platysma were divided, and a subplatysmal dissection was made above the incision up to the level of the thyroid cartilage. The fascia between the strap muscles was divided, and the anterior surface of the thyroid gland was exposed. After resection of the pyramidal lobe, a pair of subdermal electrodes (length, 12.0 mm; diameter, 0.4 mm; Medtronic, Jacksonville, FL) was inserted into the subperichondrium of the thyroid cartilage lamina on both sides (34). The thyroid cartilage electrodes were connected to the nerve integrity monitoring (NIM). The elicited EMG signals with electrode leads were documented as channels 1 and 2 simultaneously. The NIM system generated stimuli with a time window set to 50 ms and an amplitude scale set to 0.2 mV/division. The pulsed stimuli were 100 μs in duration and 4 Hz in frequency. Event capture was activated at a threshold of 100 μV. The intraoperative standardized IONM protocol routinely followed the departmental guidelines. The highest EMG amplitudes were recorded. V_1_ signal represents vagal stimulation before dissection, R_1_ signal represents RLN stimulation at first identification, R_2_ signal represents RLN stimulation after complete dissection, and V_2_ signal represents repeat vagal stimulation after resection of the thyroid.

### Outcome Measures

The primary outcomes of this study were the quality of the IONM and EMG amplitude of the V_1_ signal. The quality of IONM was measured using the obtained V_1_ amplitude. Satisfactory and weak signal was defined as a V_1_ amplitude of >500 μV and <500 μV, respectively. The secondary outcome was the quality of surgical relaxation. The quality of relaxation was assessed by the number of free periods without any intraoperative limb movement, coughing, and swallowing events. The data of NMB, anesthesia depth, hemodynamics, postoperative adverse events, and surgical outcomes were also recorded and analyzed. All patients received preoperative and postoperative video recordings of vocal cord mobility by flexible laryngofiberoscopy. When asymmetric cord movement was found postoperatively, a comparison with the preoperative recording was performed.

### Statistical Analysis

Continuous data were presented as mean ± standard deviation (SD) values, and nominal data are presented as number (%). The distribution of variables was tested using the Kolmogorov–Smirnov test. Statistical analysis of continuous variables with normal distribution between groups was compared using the unpaired t-test, while continuous variables without normal distribution were compared using the Mann–Whitney U test. All statistical tests were two-tailed. Categorical nominal variables were analyzed using the chi-square or Fisher exact test. Statistical significance was set at *P* < 0.05.

The sample size estimation was based on two similarly designed studies. To ensure adequate power for the study, a minimal sample size of 40 patients was used to measure the effect of 0.5 mg/kg sugammadex according to a previous study (31). One previous study showed satisfactory nerve monitoring (V_1_ >500 µV) in 90% of patients when the vagus nerve was monitored after delivery of 1 mg/kg sugammadex (32). To establish a non-inferiority study in which 0.25 mg/kg sugammadex would not be less satisfactory, the case number required for the study was 38 patients per group with a 10% non-inferiority margin, an alpha of 0.05, and a beta of 0.2.

## Results

A total of 116 patients (24 men and 92 women; aged 21–88 years) were included in this study (**Figure 1**). Detailed patient characteristics are shown in **Table 2**. All tracheal intubations were successful after the first attempt, and no intubation-related upper airway trauma was noted. There was no difference between the two groups in terms of demographic data, physical status, disease diagnosis, vasopressor use, and surgical relaxation. The number of patients with complete surgical relaxation was significantly lower after sugammadex injection [71 (93.4%) to 55 (72.3%) in group I, *P* < 0.01, vs. 39 (97.5%) to 33 (82.5%) in group II, *P* = 0.03]. Overall, 193 nerves were at risk, and only one nerve had intraoperative loss of signal. This was a cancer patient who had postoperative temporary RLN palsy who recovered after 4 weeks.

**Table 2:** Patient characteristics of 116 patients receiving monitored thyroidectomy.

	Group I	Group II	*P*
	(n = 76)	(n = 40)	value
Female gender	62 (81.5%)	30 (75%)	0.71
Age, mean (SD), years	51.2 (13.4)	55.6 (12.6)	0.82
Weight (kg)	60.0 (11.5)	57.8 (9.9)	0.29
Height (cm)	159.8 (7.7)	157.7 (7.3)	0.14
BMI (kg/m^2^)	23.4 (3.6)	23.2 (3.5)	0.79
ASA status			
I	2 (2.6%)	1 (2.5%)	0.89
II	56 (73.7%)	31 (77.5%)	
III	18 (23.7%)	8 (20%)	
Diagnosis			0.75
Cancer	30 (39.5%)	16 (42.5%)	
Benign	46 (60.5%)	24 (57.5%)	
Vasopressor	7 (9.2%)	5 (12.5%)	0.58
Complete relaxation*			
Before sugammadex	71 (93.4%)	39 (97.5%)	0.35
After sugammadex	55 (72.3%)	33 (82.5%)	0.23
Nerve at risk (n)	122	71	
RLN signal loss^#^	1 (0.8%)	0 (0%)	0.44
Temporary palsy	1 (0.8%)	0 (0%)	0.44
Permanent palsy	0 (0%)	0 (0%)	1.0

ASA status, American Society of Anesthesiologists Physical Status classification system; BMI, body mass index (calculated as weight in kilograms divided by height in meters squared); *Without any one event of limb movement, coughing, or swallowing; ^#^signal loss was defined as an EMG amplitude decrease of more than 50% of the baseline value.

Key time intervals for anesthesia, operation, and neuromonitoring did not differ significantly between the groups (**Table 3**). The average time from anesthesia induction (rocuronium) to sugammadex injection was 46.0 (± 9.1) min in group I and 43.4 (± 9.3) min in group II (*P* = 0.14). The average time from the surgeon’s request to administer sugammadex to initial vagal stimulation was as short as 5.4 (± 2.1) min in group I and 5.1 (± 2.1) min in group II (*P* = 0.48).

**Table 3:** Time interval of procedures and quality of intraoperative neural monitoring comparison of neuromuscular blockade degree, neural monitoring recordings, and postoperative adverse events.

	Group I	Group II	*P*
	(n = 76)	(n = 40)	value
**Time interval**			
Anesthesia to skin incision	23.8 (7.2)	22.2 (7.3)	0.26
Skin incision to sugammadex	22.2 (6.2)	21.2 (5.4)	0.38
Sugammadex to V_1_	5.4 (2.1)	5.1 (2.2)	0.48
Sugammadex to V_2_	27.6 (12.7)	31.4 (12.6)	0.12
**V_1_ amplitude**			0.23
<500 μV	1 (1.3%)	2 (5%)	
>500 μV	75 (98.7%)	38 (95%)	
**V_1_ stimulation**			
EMG amplitude (μV)	1929 (806)	1616 (939)	0.06
TOF ratio (%)	36 (28)	30 (32)	0.29
**V_2_ stimulation**			
EMG amplitude (μV)	2084 (972)	1868 (1070)	0.27
TOF ratio (%)	73 (24)	76 (19)	0.53

V_1_, initial vagal stimulation; V_2_, final vagal stimulation; EMG, electromyography; TOF, train of four.

With respect to IONM quality, all patients showed a positive V_1_ signal after sugammadex. The V_1_ EMG amplitude was greater than 500 μV in most patients. There was no significant difference between the groups (satisfactory/weak: 75/1 in group I and 38/2 in group II, *P* = 0.23). The EMG amplitude and NMB degree were compared at V_1_ and V_2_ stimulations between the two groups. At the V_1_ time point, group I demonstrated a higher EMG amplitude [1,926 (± 806) vs. 1,616 (± 939), *P* = 0.06] and a higher TOF ratio [36 (± 28) vs. 30 (± 32), *P* = 0.29] than group II, but the difference was not statistically significant (**Table 3**). Both the mean EMG amplitude and TOF ratio at the V_2_ time point were comparable between the two groups (**Table 3**).

## Discussion

The present results revealed that both 0.5 mg/kg sugammadex for deep NMB and 0.25 mg/kg sugammadex for moderate NMB could be feasible for monitored thyroidectomy. Both groups were comparable in terms of surgical relaxation quality in terms of freedom from intraoperative limb movements, coughing, and swallowing. To the best of our knowledge, this is the first report evaluating the effects of sugammadex titrated according to the degree of NMB for IONM during thyroid surgery.

Two fundamental elements of successful neuromonitoring are a proper recording of electrode position and NMB recovery. With respect to electrode position, in this study, we used a pair of outer thyroid cartilage electrodes to ensure stable EMG signals (34–39) instead of electrodes of an EMG endotracheal tube, which was susceptible to rotational or depth changes (18, 38, 40). Regarding NMB management, several feasible regimens have been proposed to facilitate tracheal intubation and functional IONM (8). Sugammadex has gained increasing popularity in IONM during thyroid surgery. The reported dose of sugammadex that was effective for IONM ranged from 0.5 to 2.0 mg/kg (28, 29, 31, 32, 41, 42). There is a wide variation between patients in the spontaneous recovery time from rocuronium-induced NMB group. Hence, precise and timely titration of sugammadex dose according to different NMB doses could be more effective and practical during IONM.

Sugammadex at 0.5 mg/kg was reported to achieve a high EMG amplitude of 1,214 (± 623) μV at V_1_ stimulation during thyroidectomy (31). Complete and early reversal of NMB was very effective for IONM, providing surgical relaxation. In clinical observations, vigorous movements might occur immediately after a dose of 1 or 2 mg/kg sugammadex. Chai et al. (32) also demonstrated that sugammadex at 2 mg/kg was associated with more bucking than 1 mg/kg (35% vs. 14%) during thyroid surgery with IONM. Since different sugammadex doses provided comparable high-quality EMG signals, this modified NMB protocol attempted to explore minimal sugammadex doses that could allow high-quality IONM signals. In a previous report, sugammadex at 0.5 mg/kg was effective for deep NMB, and sugammadex dose at 0.25 mg/kg provided moderate NMB or recovery. A reduction in sugammadex dose for a lower degree of NMB was found to be effective for high-quality IONM (V_1_ amplitude >500 μV) in 98.7% (75/76) of patients. Although there was one patient with a V_1_ signal of <500 μV (445 μV), typical EMG waveforms can be easily observed, and IONM was successfully performed without difficulty.

In addition to the dose of sugammadex, the timing of administration plays a key role in NMB management. Sugammadex has been developed to selectively bind to amino-steroidal NMBAs (i.e., rocuronium) selectively (43, 44). Sugammadex provides rapid and effective reversal of rocuronium-induced NMB not only for residual NMB after extubation but also for high-quality IONM signals during surgery. When considering the rapid time of onset, the interval between V_1_ and sugammadex in this protocol was modified to be as short as possible. This study employed a team approach during IONM, wherein the anesthesiologist administered sugammadex per the surgeon’s request after exposing the anterior surface of the thyroid gland and preparing for initial (V_1_) vagal stimulation. The overall interval from sugammadex administration to V_1_ stimulation was 5.32 (± 2.2) min.

There is a lack of consensus regarding sugammadex timing before vagal stimulation; several time points reported for administration were as follows: immediately after tracheal tube fixation, at skin incision, at 10 min after skin excision, and at exposure and identification of the vagus nerve. The interval from sugammadex to V1 stimulation was between 3 and 32 min (9, 12, 13, 22). In a selective protocol, sugammadex was used after V_1_ stimulation when the EMG amplitude was absent or below 100 μV. However, a low initial V_1_ amplitude limits the application of the IONM troubleshooting algorithm in case of signal loss (10). Sugammadex timing may affect the reversal of NMB. In this study, the mean TOF ratio was 30% at V_1_ stimulation after sugammadex 0.5 mg/kg was administered before V_1_ stimulation. In our previous report, the TOF ratio was 59% after sugammadex 0.5 mg/kg was administered 10 min after starting the operation (12). Both regimens were able to present comparable high-quality IONM signals during thyroid surgery.

There are several advantages to this surgeon-centered NMB degree titrated sugammadex protocol for monitored thyroidectomy. First, sugammadex titration based on NMB degree provided two effective rocuronium doses (0.6 mg/kg) to provide an excellent condition for tracheal intubation in most patients (9, 12). Second, sugammadex administration approximately 5 min before V_1_ stimulation ensured an adequate onset time to reverse NMB. The protocol maximizes the sugammadex-free interval to avoid bucking or movement resulting from a lower degree of NMB (13). Finally, regarding IONM quality, a high EMG amplitude was noted in 97.4% (113/116) of patients. An initial high-quality V_1_ signal is crucial to the signal loss algorithm because the initial EMG amplitude is a standard reference to be compared (1, 5, 45, 46) and to detect imminent nerve stress or injury (11, 12, 47–50).

In addition to the application of sugammadex, several alternative methods could be used for IONM during thyroid surgery. Since sugammadex is not available in all institutes because of its high cost, dose titration of neuromuscular blocking agents might also enable tracheal intubation and surgical relaxation. In a review of NMB management for IONM without using sugammadex, regimens including relaxant-free, succinylcholine, and low to standard dose of rocuronium (0.3~0.6 mg/kg) were all feasible with some clinical limitations (24, 27).

This study had several limitations. First, the study design lacked patient randomization and blinding regarding the administered sugammadex dose because of grouping based on the degree of NMB. Patients in both groups showed comparable demography, disease, and procedure profiles. Second, there might be a selection bias due to the high cost of sugammadex. This was a self-payment regimen; thus, patients who could afford sugammadex and had a high socioeconomic status were included. Third, this protocol was limited by the NMB monitoring equipment in addition to sugammadex. The NMB degree was measured by the TOF ratio generally *via* acceleromyography or kinemyography devices in Taiwan. However, both devices and sugammadex are not easily available for every institute. Finally, caution should be exercised when interpreting the outcomes in this trial. The outcomes were based on the close cooperation between the surgery and the anesthesia team with much clinical and laboratory experience in thyroid surgery with IONM. The IONM outcomes may be influenced by team members in various clinical backgrounds.

## Conclusions

Before vagal stimulation, this surgeon-centered sugammadex protocol according to NMB degree allowed high IONM quality and adequate surgical relaxation. Both sugammadex 0.5 mg/kg for deep NMB and 0.25 mg/kg for moderate NMB or less showed comparable high EMG amplitude at V_1_ stimulation. Sugammadex titration for reversal of NMB prevented undetectable or markedly low EMG amplitudes whenever nerve stimulation was required during thyroidectomy. Moreover, the flexibility of sugammadex administration timing based on the surgical procedure was also feasible for IONM during thyroid surgery. These initial positive results warrant further randomized controlled trials.

## Data Availability Statement

The raw data supporting the conclusions of this article will be made available by the authors without undue reservation.

## Ethics Statement

The studies involving human participants were reviewed and approved by the institutional review board of Kaohsiung Medical University Hospital, Kaohsiung Taiwan. Written informed consent for participation was not required for this study in accordance with the national legislation and institutional requirements.

## Author Contributions

C-WW had full access to all the data in the study and takes responsibility for the integrity of the data and the accuracy of the data analysis. Y-WK and C-WW contributed equally to this work. Concept and design: I-CL and C-WW. Acquisition, analysis, or interpretation of data: I-CL, C-DH, P-YC, GD, YJC, C-WW. Drafting of the article: I-CL, F-YC, Y-WK, C-WW. Critical revision of the article and final approval: All authors. Statistical analysis: I-CL, F-YC, Y-CL, C-WW. Obtained funding: I-CL, T-YH, C-WW. Administrative, technical, or material support: S-HW, T-YH, Y-CL, H-YK, and F-YC. Supervision: Y-WK and C-WW.

## Funding

This study was supported by grants from the Kaohsiung Municipal Siaogang Hospital (H-109-001, Kmhk-110-08) and the Ministry of Science and Technology, Taiwan (MOST 109-2314-B-037-059).

## Conflict of Interest

The authors declare that the research was conducted in the absence of any commercial or financial relationships that could be construed as a potential conflict of interest.

## Publisher’s Note

All claims expressed in this article are solely those of the authors and do not necessarily represent those of their affiliated organizations, or those of the publisher, the editors and the reviewers. Any product that may be evaluated in this article, or claim that may be made by its manufacturer, is not guaranteed or endorsed by the publisher.
